# Pretreatment Beck Depression Inventory score is an important predictor for Post-treatment score in infertile patients: a before-after study

**DOI:** 10.1186/1471-244X-5-25

**Published:** 2005-05-24

**Authors:** Afsaneh Khademi, Ashraf Alleyassin, Marzieh Aghahosseini, Fatemeh Ramezanzadeh, Ali Ahmadi Abhari

**Affiliations:** 1 Infertility Ward, Shariati Hospital, Tehran University of Medical Sciences, North Kargar Street, Tehran, 14114, I R Iran; 2 Vali-e-Asr Reproductive Health Research Center, Vali-e-Asr Hospital, Emam Khomeini Hospital, Tehran University of Medical Sciences, Keshavarz Blvd, Tehran, 14194, I R Iran; 3 Roozbeh Hospital, Tehran University of Medical Sciences, South Karger Street, Tehran, 13185, I R Iran

## Abstract

**Background:**

The experience of infertility can be extremely stressful. Some of the risk factors for depression in infertility are being female, repeated unsuccessful treatment cycles or a 2 to 3 year history of infertility, low socioeconomic status, foreign nationality, lack of partner support, life events and previous depression. In this study, we analyzed the Beck Depression Inventory score at the beginning and the end of infertility treatment, to determine which factors may influence the BDI score after treatment of infertility.

**Methods:**

In a before-after study, in a university-affiliated teaching hospital, 251 women who had been visited for assisted reproductive technology infertility treatment participated in the study. BDI score was assessed before and after treatment of infertility.

**Results:**

The mean BDI score rose after unsuccessful treatment and dropped after successful treatment. Those with lower education levels had a higher BDI score before treatment. BDI score after treatment was positively correlated with pretreatment BDI scoreand duration of infertility.

**Conclusion:**

BDI score after treatment was strongly connected to  the BDI score before treatment, the result of therapy and to the duration of infertility. The influence of duration of infertility on BDI score after treatment of infertility is weak. So a simple method to screen patients at risk of depression after infertility treatment is determining pretreatment BDI score and predicting the result of infertility treatment by other risk factors.

## Background

Almost 21% of the female population experience major depression in their life [1]. Twice as many females experience some form of depression when compared to male [2]. Female depression has higher risk on first onset, can last longer, and often recur [3,4]. There is established relationship between life stress and depression [5].

Infertility is a stressful event in life of human being. In a comparison to patients with other medical conditions, psychological symptoms associated with infertility are similar to those related to cancer, hypertension, and cardiac rehabilitation [6]. Infertile women, in comparison with control group, showed higher scores on the depression and anxiety scales [7,8].

Evidentially, depression has some neurobiological and hormonal mechanisms. Changes in ovarian hormones are likely necessary, but not sufficient for inducing depression [9]. The risk of relapse after treatment is 2.5 times higher in women; on the other hand, non-remission rate is similar in both sexes [10]. It appears that the sex difference cannot explain the sex-ratio relapse rate. As a result, there should be multiple factors including sex for inducing depression overall and in infertile subjects [3]. Many studies have been considered to determine risk factors of depression in infertility. In some studies, risk factors include female gender, repeated treatment cycles, unsuccessful treatments, low socioeconomic state, foreign nationality, lack of partner's support for women, life events for female, previous depression, and 2 to 3 years history of infertility [8,11,12]. However, some of these factors such as short period of infertility and husband cooperation have not been confirmed in other studies [12,13]. The above-mentioned studies were designed as cross-sectional studies. So far, there is no prospective cohort or before-after study to find predictors of depression in infertile patients after treatment except a single one [14]. Lok et al showed that severity of depression following a failed treatment was positively associated with the duration of infertility. However, the post-treatment BDI scores were not associated with type of infertility treatment received, age, education, cause of infertility and number of previous treatments received [14].

Screening depression is a difficult task. The reason being is that there are numerous questionnaires for interpretation of psychological symptoms. Beck Depression Inventory (BDI) was developed and revised by Beck et al [15,16]. This 21-item self-report questionnaire was intended to assess the severity of current depressive symptomatology in the psychiatric population. It is written on a fifth or sixth-grade reading level. It requires minimal time and no special training to administer. The BDI has been used, extensively, in clinical diagnosis and research [15,16]. Versions in other languages are confirmed and used, as well [17-19]. In our study, Persian version of BDI has been employed.

We analyzed Beck Depression Inventory score at the beginning and at the end of treatment cycle to determine which factors may influence the score of BDI, after treatment of infertility.

## Methods

Between April 2003 and 2004, 350 infertile women, whom had been visited in the Infertility Ward of Shariati Hospital in Tehran, participated in a prospective before-after study.

Exclusion criteria were out of Tehran residency, definite diagnosis of mood disorders (with or without current treatment), medications reportedly associated with depression (e.g. steroidal contraceptives), and medical conditions associated with depression such as thyroid dysfunction and diabetes mellitus [20]. All subjects signed informed consent. Demographic data collected included age, education, type of infertility, duration of infertility, cause of infertility, and history of previous treatment (expressed as number). Candidates were asked to complete BDI (BDI1) in their first visit, during waiting periods, at the outpatient clinic. The questionnaire was completed by an assistant for uneducated subjects. Accordingly, BDI score was calculated.

Index score of ≤9 is considered to be within normal range, a score of 10 to 15 shows minimal depressive symptomatology, a score of 16–31 points toward mild depression, a score of 32–47 is in favor of moderate depression and a score of >47 indicates severe depression [16].

Due to rules of ethics, 10 patients who had the score of 47 or above were informed about their condition. Eight of these 10 patients were excluded from study since it was essential for them to seek psychiatric consultation as soon as possible. We continued the study with 342 patients. The treatment consisted of either intrauterine insemination (IUI) or assisted reproductive technology (ART). IUI candidates were excluded from the study. Therefore, the sample size was 257. At day 16 after embryo transfer, a positive result of treatment was defined as beta hCG (β-hCG) ≥200 IU/ml. One to Three weeks after receiving result of β-hCG, patients were asked to complete the second BDI (BDI2).

This study was approved by ethics committee of Tehran University of Medical Sciences. The statistical analysis was performed using the Statistical Package for Social Sciences, version 10.0 (SPSS Inc., Chicago, IL). The primary outcome measure was the score of BDI after treatment. Data was expressed as means +/-SD and percentile of total. The tests being used were Wilcoxon Signed Ranks test, Mann-Whitney test, one way ANOVA, multiple regression analysis, and chi-square test.

## Results

A total number of 257 women were entered into the study after completing BDI1. Six (2.3%) patients refused answering BDI2. As a result, there were 251 paired complete questionnaires.

Demographic characteristics of patients are given in Table 1. We divided the patients into four groups based on cause of infertility. The prevalence was lowest in unexplained infertility (10%) and highest in female infertility (43%). The education level was divided into six groups. A large number of participants were at intermediate education level (35.1%) and a few of them were uneducated (2.4%). The number of previous attempts for treatment of infertility (IUI and/or ART) defers from 0 (24.7%) to 11 times (0.4%). Positive result of β-hCG was achieved in 61 patients (24.3%) (Table 1).

**Table 1:** Demographic characteristics of subjects

Characteristic		No.	Mean(± SD) or %
Age (y)		251	28.9 ± 5.5
Duration of infertility (y)		251	6.9 ± 4.5
No. of previous treatment		251	1.3 ± 1.4
			
Education	No education	6	2.4
	Primary school	36	14.3
	Junior school	60	23.9
	High school	12	4.8
	Intermediate education	88	35.1
	University	49	19.5
			
Type of infertility	Primary	230	91.6
	Secondary	21	8.4
			
Cause of infertility	Male	86	34.3
	Female	108	43
	Combined	32	12.7
	Unexplained	25	10
			
Result of β-hCG	Positive	61	24.3
	Negative	190	75.7

The differences of somatic variables were analyzed between pregnant and non-pregnant subgroups. Pregnant subjects were younger (*P *< 0.000), and had shorter duration of infertility (*P *= 0.005). There were no statistically significant difference in terms of type of infertility, education, cause of infertility, mean number of previous treatment, and mean score of BDI1 (Table 2).

**Table 2:** Comparison of demographic characteristics between pregnant and non-pregnant subjects

		Pregnant (n = 61)	Non-pregnant (n = 190)	*P*
Mean age (y)		26.6 ± 4.2	29.6 ± 5.7	<.000
Duration of infertility (y)		5.5 ± 3.7	7.4 ± 4.6	.003
Primary infertility (%)		58 (95.1)	172 (90.5)	NS
Cause of infertility (%)	Male	22 (36.1)	64 (33.7)	NS
	Female	27 (44.3)	81 (42.6)	
	Both	7 (11.5)	25 (13.2)	
	Unknown	5 (8.2)	20 (10.5)	
				
Education (%)	No education	0	6 (3.2)	NS
	Primary school	6 (9.8)	30 (15.8)	
	Guiding school	11 (18)	49 (25.8)	
	High school	3 (4.9)	9 (4.7)	
	Completed high school	29 (47.5)	59 (31.1)	
	University	12 (19.7)	37 (19.5)	
				
Previous treatment		1.2 ± 1.4	1.3 ± 1.4	NS
BDI1 score		14.6 ± 11.3	14.5 ± 9.8	NS

The BDI score at the beginning of the study (BDI1) was within normal range (0–15) in 61% of patients. In 31% the score was 16–31, suggestive of mild depression, and only 8% of the women had BDI score higher than cutoff score (≥32), suggestive of moderate and severe depressive symptoms.

After knowing the results of β-hCG, BDI score changed. After treatment, 47% of the whole sample was within normal range. In pregnant and non-pregnant subjects, normal score of BDI2 was found 95.1% and 31.6%, respectively. The difference in percentile between pregnant and non-pregnant patients were statistically significant (*P <*.000).

Our main outcome was evaluation of factors that can influence BDI2 score. When BDI2 score was considered as dependent variable, in a multiple regression analysis, it was positively correlated with BDI1 score (r = 0.61, *P *< .000) and duration of infertility (r = 0.15, P < .000). Negative correlation was found between BDI2 score and result of therapy (r = - .51, *P *= .000). Eighty six percent of variation of BDI2 score could be predicted by the above three variables. There was no correlation between BDI2 score and age, education, number of previous treatment, and type of infertility.

The mean score of BDI2 was evaluated in relation to cause of infertility. The mean score in male factor was less than other groups. The difference between groups was statistically significant (Table 3).

**Table 3:** Mean score of BDI1 and BDI2 in relation to cause of infertility

Cause of infertility	Mean of BDI1	Mean of BDI2		
Male	11.2 ± 8.9	14.6 ± 11.3	*P *< .000*	Z = -4.168
Female	15.8 ± 11	21.1 ± 13.2	*P *< .000*	Z = -4.745
Combined	16.7 ± 9.2	21.9 ± 14.3	*P *= .006*	Z = -2.735
Unknown	15.2 ± 9.5	19.9 ± 10.5	*P *= .02*	Z = -2.303
	*P *= .006**	*P *= .002**		
Total	14.3 ± 10.2	18.9 ± 12.8	*P *< .000	Z = -7.196

*: difference between variables in row

**: difference between variables in columns

Mean of BDI2 score rose after unsuccessful treatment and dropped after a successful treatment (Table 4).

**Table 4:** Comparison of mean BDI1 and BDI2 in pregnant and non-pregnant subjects

	pregnant	Non-pregnant	*P*
BDI1 (mean ± SD)	13.6 ± 11.3	14.5 ± 9.8	.28 (Z = -1.079)
BDI2 (mean ± SD)	6.2 ± 5.4	22.9 ± 11.8	<.000 (Z = -9.180)
*P*	<.000 (Z = -5.845)	<.000 (Z = -11.481)	

We observed the factors that affect BDI2 score in pregnant and non-pregnant groups, separately. In pregnant subjects, BDI2 score was positively correlated with BDI1 score (r = 0.82, *P *< .000). In non-pregnant patients, BDI2 score was positively correlated with BDI1 score (r = 0.8, *P *< .000), and duration of infertility (r = 0.17,*P *= .001).

We studied BDI1 score, as well. In a multiple regression analysis, BDI1 score was negatively correlated with education (r = -0.26, *P *< 0.000). There was no association between BDI1 score and age, duration of infertility, previous fertility, and number of previous cycles of treatment. Only 26% of variation of BDI1 could be predicted by the factor of education.

In subgroups of infertility cause, the mean score of BDI1 in male factor was less than other groups. The difference between the groups was statistically significant (Table 3).

## Discussion

The purpose of this study was to describe the subgroups of infertile women at risk of depression after treatment of infertility. This study can be valuable because it is using a self-reported inventory that differs in both cost and time from psychiatric structured interview. Nevertheless the shortcomings of this inventory, it has been widely used in research. Sensitivity and specificity of Beck Depression Inventory is not high but is reasonable. Beck et al suggested that a score greater than 9 points to depression symptomatology [16]. BDI score were also categorized in subgroups. The score of 9 and less shows normal range, a score of 10–15 indicates at least mild to moderate depressive symptoms, and a score of 16 and above indicates clinical depression [21]. We chose the second version since it screens subgroups of depression more precisely.

In this study, the prevalence of depressive symptomatology as assessed by BDI1 score ≥ 16 was 39%, while the prevalence of moderate to severe depression(BDI ≥ 32) In an interesting study, the questionnaires were sent through internet to estimate degree of some psychological characteristics such as depression. Based on this study, authors found out that more than one quarter of patients could be considered moderately or severely depressed [22]. In screening depression in pregnancy, researchers chose a score greater than 9 and 43% of their population scored above this cutoff. As they designated BDI score greater than 15 as abnormal, nevertheless, the score exceeded the cutoff value in 19% of patients. In that study, a cutoff value greater than 15 yields a sensitivity of 0.83, a specificity of 0.89, a positive predictive value of 0.50, and a negative predictive value of 0.98 [21]. One of the best ways to determine the prevalence of a disease or symptom in relation to a risk factor is to compare a case group with a well-chosen control group. Domar et al showed that the prevalence of depression was 25.8% in infertile women compared with 13.2% in women who were waiting for a routine gynecologic examination [8]. These percentiles were reported to be 36.7% compared to 18.4% in another study [23]. Therefore, it is important that self-report questionnaires used for screening a disease should have a cutoff point reaffirmed either by a gold standard test or control group.

The main outcome of this study was determining factors that may influence BDI2. The score after treatment (BDI2) was correlated with the score of BDI before treatment and duration of infertility and with the result of therapy.

Hence, Table 4 shows no statistically significant difference between mean score of BDI1 in the two groups, we assume that the factors affecting BDI1 in the pregnant group and those in the non-pregnant group are the same.

As BDI1 had a strongest affect on BDI2, a regression analysis considering BDI1 as a dependent variable was performed. It was found out that BDI1 score decreases as the level of education increases. In a stepwise regression, we failed to show any other factors to have relation with BDI1 score. There have been studies in which correlation between BDI score and demographic characteristics were evaluated. Demyttenaere et al showed that age, duration of infertility, and numbers of previous IVF attempts are factors affecting BDI score, but there was no data about level of education [24]. On the other hand, Beutel et al reported the correlation between education and depression (Spearsman correlation = -0.15 *P *< .05) [11]. This inverse relationship between education and BDI1 score was considerable in our study (r = -0.26). This relationship can be explained by cultural views. It seems that high-educated people have objectives other than fertility to focus on.

Data about age and risk of depression is not conclusive. Some studies have reported associations been them [11,24,25], while other studies could not show any relationships between these two [14,26]. We suggested that other variables could act as intermediate variables between age and BDI1. Because of the strength of theses variables, the effect of age was hidden in multivariate analysis.

The second important factor was the result of therapy. Apparently, subjects who are at risk of depression are more prone to develop depression at the time of exposure to life events.

The third factor that had influence on BDI2 was duration of infertility. The association of BDI score after treatment with duration of infertility in subgroup with failed treatment was showed in another study [14]. In our study, BDI2 is associated positively with duration of infertility. However, the strength of this association is low (r = 0.15). It seems that such a low influence cannot play a significant role in practice. The assumption on whether the association between duration of infertility and post-treatment BDI score is a straight association or depends on intermediate variables such as number of treatment failure experiences needs more studies [27].

The perception of woman about the cause of infertility may change her BDI score. Pressure on infertile women with female factor infertility is high. The risk of depressive symptomatology is lower when a woman thinks that the problem is male factor. Combined factor indicates a severe problem, so it is reasonable that BDI score rises by this diagnosis (Table 3). This type of cultural view has been showed in countries with family-based societies [28].

We emphasize that a number of meaningful relationships were introduced in other studies which were not mentioned here. Thus, in prospective studies other variables should be included and other more sensitive tools should be used.

The other limitation of this study was using BDI for uneducated subjects that might induce a bias. It is clear that designing studies using interviews instead of questionnaires will be more valuable.

## Conclusion

Our findings suggest a plan for screening women who are prone to depression after infertility treatment. It is obvious that there is no significant difference among them before treatment (Table 2). Answering the following two simple questions can conduct the physician to screen these women.

1. Is my patient at risk of depression (due to BDI1 score) at the beginning of the treatment?

2. What will be the result of therapy?

Therefore, the physician can predict subjects who may need psychological supportive programs at the beginning of treatment cycles.

## Competing interests

The author(s) declare that they have no competing interests.

## Authors' contributions

AK, AA and MA drafted the manuscript. AK performed the statistical analysis. AK, AAA and FR participated in the study design and coordination. All authors read and approved the final manuscript.

## Pre-publication history

The pre-publication history for this paper can be accessed here:



## References

[B1] Yonkers KA, Chantilis SJ (1995). Recognition of depression in obstetric/gynecology practices. Am J Obstet Gynecol.

[B2] Desai HD, Jann MW (2000). Major depression in women: a review of the literature. J Am Pharm Assoc (Wash).

[B3] Kessler RC (2003). Epidemiology of women and depression. J Affect Disord.

[B4] Lucht M, Schaub RT, Meyer C, Hapke U, Rumpf HJ, Bartels T, von-Houwald J, Barnow S, Freyberger HJ, Dilling H, John U (2003). Gender differences in unipolar depression: a general population survey of adults between age 18 to 64 of German nationality. J Affect Disord.

[B5] Paykel ES (2001). Stress and affective disorders in humans. Semin Clin Neuropsychiatry.

[B6] Domar AD, Zuttermeister PC, Friedman R (1993). The psychological impact of infertility: a comparison with patients with other medical conditions. J Psychosom Obstet Gynaecol.

[B7] Wischmann T, Stammer H, Scherg H, Gerhard I, Verres R (2001). Psychosocial characteristics of infertile couples: a study by the 'Heidelberg Fertility Consultation Service'. Hum Reprod.

[B8] Domar AD, Broome A, Zuttermeister PC, Seibel M, Friedman R (1992). The prevalence and predictability of depression in infertile women. Fertil Steril.

[B9] Bloch M, Schmidt PJ, Danaceau M, Murphy J, Nieman L, Rubinow DR (2000). Effects of gonadal steroids in women with a history of postpartum depression. Am J Psychiatry.

[B10] Kuehner C (1999). Gender differences in the short-term course of unipolar depression in a follow-up sample of depressed inpatients. J Affect Disord.

[B11] Beutel M, Kupfer J, Kirchmeyer P, Kehde S, Köhn FM, Schroeder-Printzen I, Gips H, Herrero HJ, Weidner W (1999). Treatment-related stresses and depression in couples undergoing assisted reproductive treatment by IVF or ICSI. Andrologia.

[B12] Kee BS, Jung BJ, Lee SH (2000). A study on psychological strain in IVF patients. J Assist Reprod Genet.

[B13] Chiba H, Mori E, Morioka Y, Kashiwakura M, Nadaoka T, Saito H, Hiroi M (1997). Stress of female infertility: relations to length of treatment. Gynecol Obstet Invest.

[B14] Lok IH, Lee DT, Cheung LP, Chung WS, Lo WK, Haines CJ (2002). Psychiatric morbidity amongst infertile Chinese women undergoing treatment with assisted reproductive technology and the impact of treatment failure. Gynecol Obstet Invest.

[B15] Beck AT, Ward CH, Mendelson M, Mock J, Erbaugh J (1961). An inventory for measuring depression. Arch Gen Psychiatry.

[B16] Beck A, Steer R, Garbin M (1988). Psychometric properties of the beck Depression Inventory: Twenty-five years of evaluation. Clin Psychol Rev.

[B17] Tekbas OF, Ceylan S, Hamzaoglu O, Hasde M (2003). An investigation of the prevalence of depressive symptoms in newly recruited young adult men in Turkey. Psychiatry Res.

[B18] Coelho R, Martins A, Barros H (2002). Clinical profiles relating gender and depressive symptoms among adolescents ascertained by the Beck Depression Inventory II. Eur Psychiatry.

[B19] Kojima M, Furukawa TA, Takahashi H, Kawai M, Nagaya T, Tokudome S (2002). Cross-cultural validation of the Beck Depression Inventory-II in Japan. Psychiatry Res.

[B20] Akiskal HG, Sadock BJ, Sadock VA (2000). Mood disorders: Clinical Features. Comprehensive Textbook in Psychiatry.

[B21] Holcomb WL, Stone LS, Lustman PJ, Gavard JA, Mostello DJ (1996). Screening for depression in pregnancy: Characteristics of the Beck Depression Inventory. Obstet Gynecol.

[B22] Epstein YM, Rosenberg HS, Grant TV, Hemenway BAN (2002). Use of the internet as the only outlet for talking about infertility. Fertil Steril.

[B23] Thiering P, Beaurepaire J, Jones M, Saunders D, Tennant C (1993). Mood state as a predictor of treatment outcome after in vitro fertilization/embryo transfer technology (IVF/ET). J Psychosom Res.

[B24] Demyttenaere K, Bonte L, Gheldof M, Vervaeke M, Meuleman C, Vanderschuerem D, D'Hooghe T (1998). Coping style and depression level in in vitro fertilization. Fertil Steril.

[B25] Guz H, Ozkan A, Sarisoy G, Yanik F, Yanik A (2003). Psychiatric symptoms in Turkish infertile women. J Psychosom Obstet Gynaecol.

[B26] Matsubayashi H, Hosaka T, Izumi S, Suzuki T, Makino T (2001). Emotional distress of infertile women in Japan. Hum Reprod.

[B27] Boivin J, Takefman JE, Tulandi T, Brender W (1995). Reactions to infertility based on extent of treatment failure. Fertil Steril.

[B28] Dyer SJ, Abrahams N, Hoffman M, van der Spuy ZM (2002). Men leave me as I cannot have children: women's experiences with involuntary childlessness. Hum Reprod.

